# Deep Sulcus Sign Revealing Occult Pneumothorax After Lumbar Spine Surgery: A Case Report

**DOI:** 10.7759/cureus.92551

**Published:** 2025-09-17

**Authors:** Hirohito Hirata, Yu Toda, Takaomi Kobayashi, Tomohito Yoshihara, Masatsugu Tsukamoto, Tadatsugu Morimoto

**Affiliations:** 1 Department of Orthopaedic Surgery, Faculty of Medicine, Saga University, Saga, JPN

**Keywords:** deep sulcus sign, lumbar burst fracture, percutaneous pedicle screws, pneumothorax, posterior spinal fixation, postoperative complication, spinal surgery

## Abstract

Pneumothorax is a rare but potentially serious complication following posterior spinal fixation. We report the case of an 80-year-old woman who developed a left-sided pneumothorax after posterior fixation with percutaneous pedicle screws (PPS) for an L1 burst fracture. The procedure was uneventful, with no significant changes in intraoperative vital signs, and the patient remained asymptomatic postoperatively. However, routine postoperative radiographs revealed a left-sided pneumothorax, indicated by a deep sulcus sign. A chest tube was promptly inserted, resulting in resolution of the pneumothorax without recurrence. Postoperative computed tomography confirmed proper pedicle screw placement without evidence of mispositioning. This case illustrates the importance of recognizing subtle radiological signs of pneumothorax in postoperative imaging, even in the absence of symptoms. Routine collaboration between orthopedic surgeons and radiologists can help detect such complications early and allow for timely intervention, which is essential to avoid adverse outcomes.

## Introduction

Osteoporotic vertebral fractures are commonly seen in the elderly population and frequently occur following low-energy trauma, such as a fall from standing height. In cases of vertebral fractures at the thoracolumbar junction, surgical stabilization may be considered when the fracture is mechanically unstable and associated with severe pain and marked restriction in daily activities, in order to relieve symptoms and prevent further collapse or neurological deterioration. Posterior fixation using percutaneous pedicle screws (PPS) is a widely accepted minimally invasive technique offering effective stabilization with reduced soft tissue disruption [1]. However, several complications associated with PPS have been reported, including screw misplacement, guidewire-related injury, vascular injury, cerebrospinal fluid leakage, and infection [2].

Although generally safe, this procedure carries potential risks, including rare thoracic complications such as pneumothorax. Because the pleura lies anatomically close to the thoracolumbar junction, surgical procedures in this region carry a potential risk of pleural injury. Postoperative pneumothorax following PPS fixation has been rarely reported, and its subtle radiographic presentation can make early diagnosis challenging. Prompt recognition is crucial to prevent life-threatening deterioration, especially when clinical symptoms are absent. This report aims to highlight the importance of careful postoperative imaging review and multidisciplinary collaboration for early detection of pneumothorax after PPS fixation.

## Case presentation

**Figure 1:**
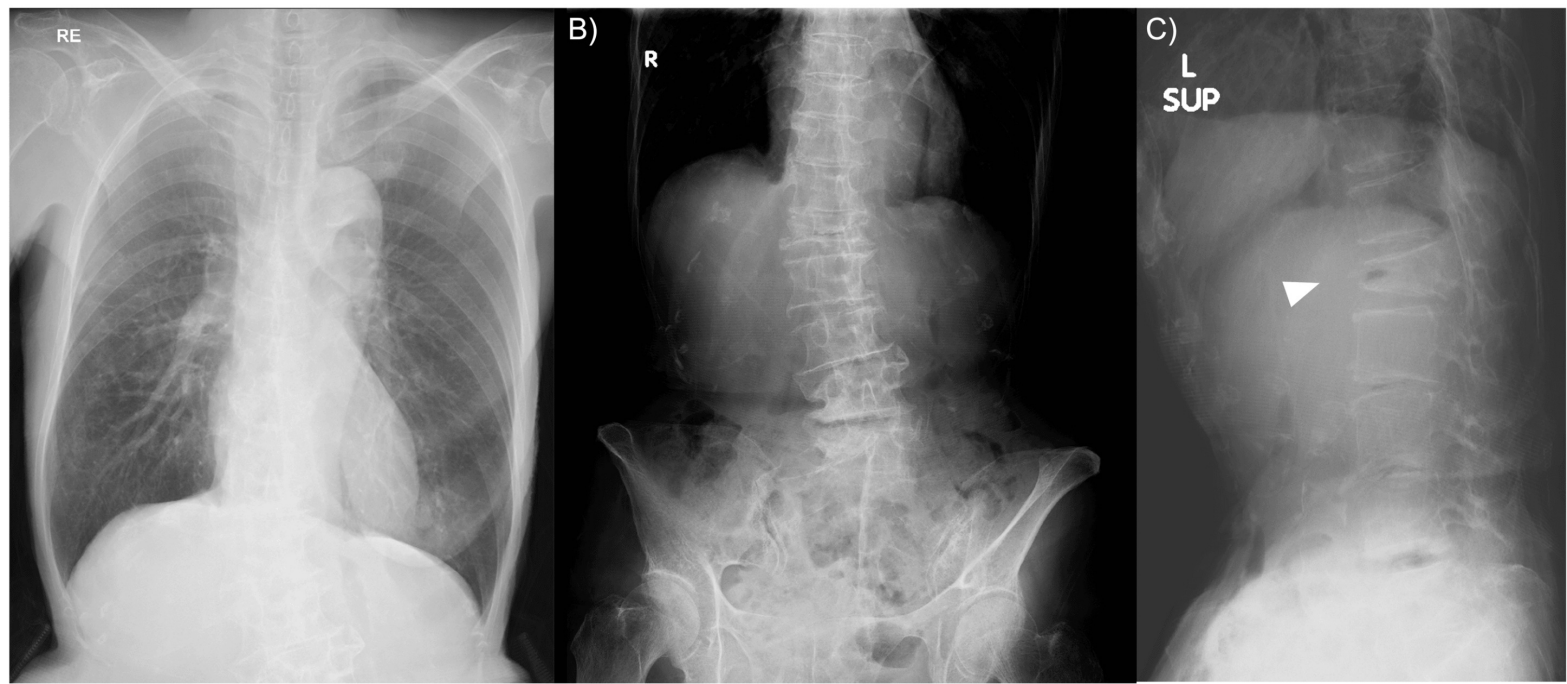
Preoperative Radiographs of the Chest and Lumbar Spine Preoperative chest radiograph (A) and lumbar spine anteroposterior (B) and supine lateral (C) radiographs showing no evidence of pneumothorax. The arrowhead in C indicates the fractured vertebra.

**Figure 2:**
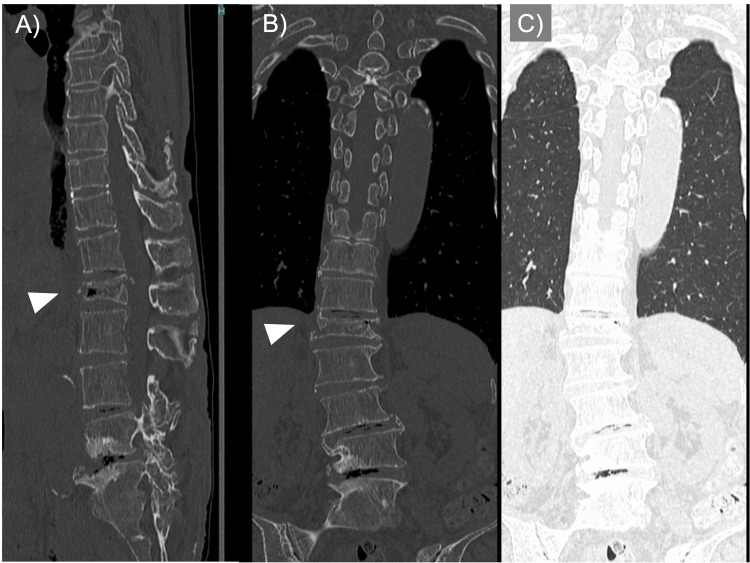
Preoperative CT Images of the Lumbar Spine and Thorax Preoperative sagittal (A), coronal (B), and coronal lung-window (C) CT images confirming the absence of pneumothorax. The arrowheads in A and B indicate the fractured vertebra.

An 80-year-old woman presented to the local hospital following a fall. She had a medical history of osteoporosis, breast cancer (on chemotherapy), and hypertension, and had been living independently and performing daily activities and gardening. On arrival, her vital signs were stable. Physical examination revealed localized tenderness and paravertebral muscle guarding at the thoracolumbar junction, with no percussion pain, no neurological deficits, and no bladder or bowel disturbances. She had no history or radiological evidence of pulmonary disease, chest trauma, or infection, and preoperative CT showed no emphysematous changes, bullae, or other lung lesions. Radiographic evaluation revealed an L1 burst fracture without spinal canal compromise (Figures 1, 2).

She initially chose conservative treatment, but follow-up radiographs about two months later showed progressive vertebral instability, and she was referred to our hospital. Given her desire for early mobilization and return to activities of daily living, the patient opted for surgical intervention.

She underwent posterior fixation using percutaneous pedicle screws (PPS) from T12 to L2, along with balloon kyphoplasty at L1. The screws were inserted under fluoroscopic guidance, using a groove entry technique at T12 and the standard PPS trajectory at L2. No obvious pleural injury due to anterior probe slippage or guidewire penetration was observed intraoperatively. The procedure lasted 46 minutes with minimal blood loss (50 ml).

**Figure 3:**
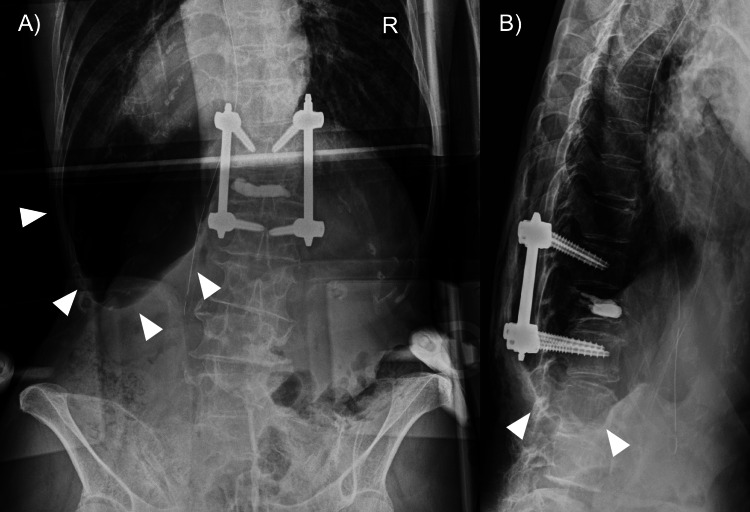
Postoperative Lumbar Spine Radiographs Showing Deep Sulcus Sign Immediate postoperative lumbar spine anteroposterior (A) and lateral (B) radiographs obtained to confirm instrumentation. Multiple arrowheads in (A) indicate a left-sided deep sulcus sign with subtle loss of vascular markings, suggesting pneumothorax.

In our institution, as part of a patient safety protocol, it is mandatory to obtain postoperative radiographs of the surgical site immediately after the procedure while the patient is still under anesthesia in the operating room. These radiographs (Figure 3) are routinely double-checked by the radiology department before the patient is extubated. In this case, the images were first reviewed by the surgical team, including the attending orthopedic surgeon, and no abnormalities, such as screw misplacement, were noted. During the radiology review performed before extubation, a left-sided deep sulcus sign was identified on the chest field of the lumbar radiograph (Figure 3), accompanied by subtle loss of vascular markings. No apparent basilar hyperlucency or other typical signs of pneumothorax were observed. At that time, the patient remained hemodynamically stable, and the nursing staff had not recorded any abnormal vital signs or respiratory symptoms.

**Figure 4:**
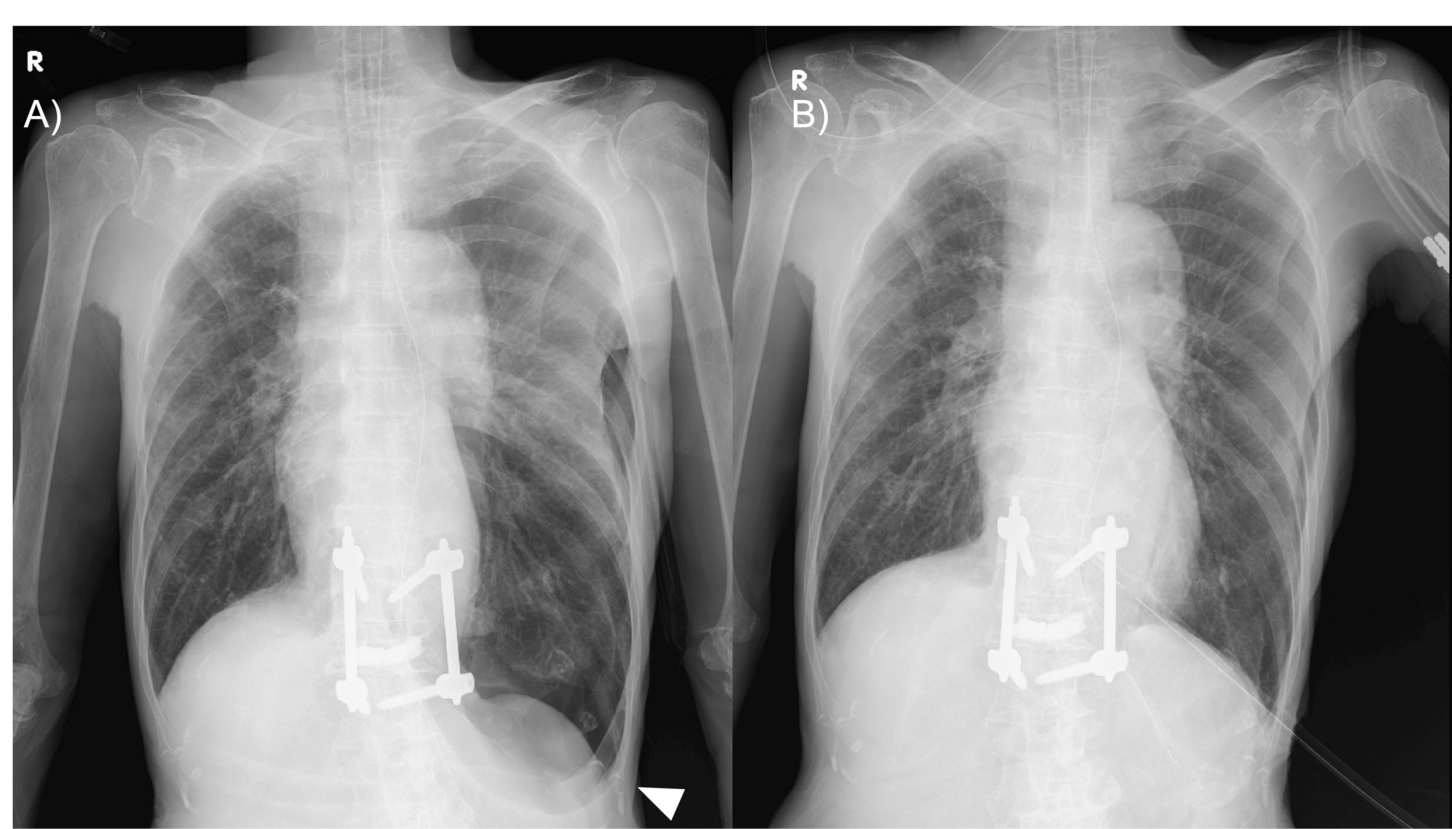
Postoperative and Follow-up Chest Radiographs Postoperative chest radiograph (A) showing a left-sided pneumothorax. Follow-up chest radiograph after chest tube treatment (B) confirms resolution without recurrence.

**Figure 5:**
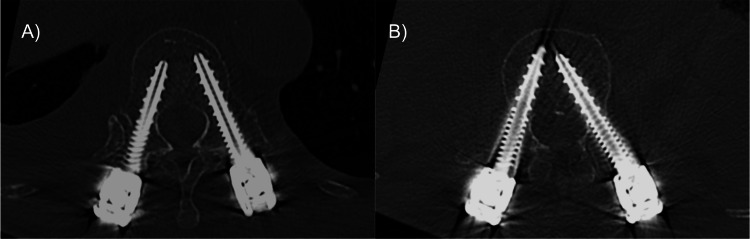
Postoperative CT Confirmation of Pedicle Screw Placement. Postoperative CT images of pedicle screw placement. CT scans showing accurate placement of pedicle screws at the Th12 (A) and L2 (B) levels with no evidence of misplacement.

Because the pneumothorax was extensive and considered to be in a tension state, a chest tube was inserted before vital signs deteriorated, even though there were no intraoperative or immediate postoperative clinical symptoms. The pneumothorax resolved without complications following chest tube insertion (Figure 4A, 4B). No active air leakage was observed, and the chest tube was removed on postoperative day 3, with no recurrence thereafter. Postoperative CT on day 7 confirmed accurate pedicle screw placement at T12 and L2, with no evidence of cortical breach or pleural injury (Figure 5A, 5B).

Following chest tube removal, the patient’s postoperative course was uneventful. Ambulation was initiated promptly, and she demonstrated steady improvement in activities of daily living. The surgical wound healed without issues, and she was transferred to a rehabilitation facility in stable condition. At the final follow-up, there was no recurrence of pneumothorax or implant-related complications, and her functional status had returned to the pre-injury level of independence.

## Discussion

Pneumothorax is a rare but clinically significant complication following posterior spinal fixation, with a reported incidence ranging from 0.2% to 0.4% [3-5]. In this case, the pneumothorax was incidentally detected via the deep sulcus sign on postoperative radiographs, despite the absence of intraoperative hemodynamic instability or respiratory symptoms [6]. The patient’s advanced age and the thoracic location of the instrumentation may have contributed to the susceptibility to this complication.

Iatrogenic pleural injury

One possible mechanism is iatrogenic pleural injury during percutaneous pedicle screw (PPS) insertion, particularly in the thoracic spine, where the pleura lies in close proximity to the entry point. The use of guidewires during screw placement increases the risk of deviation and inadvertent pleural penetration, even with fluoroscopic guidance [7].

Screw misplacement or microtrauma

Another potential mechanism is screw misplacement, which has been identified as a cause of pneumothorax in multiple reports [8]. Although postoperative CT in our case did not demonstrate obvious screw perforation, subtle cortical breaches or intraoperative microtrauma cannot be completely excluded.

Pre-existing or anesthesia-related pneumothorax

Alternatively, the patient may have had a pre-existing traumatic pneumothorax that was not detected during the initial evaluation. Thoracic burst fractures, particularly in elderly individuals, can sometimes be accompanied by minor pleural injuries. The use of positive pressure ventilation during general anesthesia may have exacerbated an otherwise clinically silent pneumothorax, enlarging it to a radiographically visible state [9].

Clinical implications

Although thoracic PPS placement using the Groove-Entry technique has been reported to provide high accuracy and a favorable safety profile [10], anatomical constraints and the proximity of the pleura require meticulous technique. Even in experienced hands, the possibility of pleural irritation or injury remains.

This case emphasizes the need for heightened postoperative vigilance, especially in thoracic spinal surgeries. The Deep Sulcus Sign, although subtle, can serve as an important early indicator of pneumothorax on supine radiographs [6]. Routine collaboration between orthopedic surgeons and radiologists is essential to identify such findings promptly.

Moreover, this case highlights the importance of considering complications beyond the surgical field. While intraoperative imaging may confirm accurate screw placement, postoperative radiographs must be reviewed comprehensively to assess for complications in adjacent structures, including the pleura and lungs.

## Conclusions

Pneumothorax is an uncommon but significant postoperative complication after posterior spinal fixation. Awareness of subtle radiological signs such as the Deep Sulcus Sign, along with prompt diagnosis and intervention, is essential to avoid delayed treatment. Routine postoperative imaging should be reviewed carefully by both radiologists and surgeons, even in asymptomatic cases.
